# Low pyrrolizidine alkaloid levels in perennial ryegrass is associated with the absence of a homospermidine synthase gene

**DOI:** 10.1186/s12870-018-1269-6

**Published:** 2018-04-06

**Authors:** Geoffrey P. Gill, Catherine J. Bryant, Mikhail Fokin, Jan Huege, Karl Fraser, Chris Jones, Mingshu Cao, Marty J. Faville

**Affiliations:** 1 Pastoral Genomics, c/o AgResearch Grasslands Research Centre, Private Bag 11008, Palmerston North, 4442 New Zealand; 2 AgResearch Grasslands Research Centre, Private Bag 11008, Palmerston North, 4442 New Zealand

**Keywords:** Pyrrolizidine alkaloids, Homospermidine synthase, Deoxyhypusine synthase, *Lolium perenne* L., Thesinine-rhamnoside, Association mapping

## Abstract

**Background:**

Pyrrolizidine alkaloids (PAs) are a class of secondary metabolites that function as feeding deterrents in a range of different plant species. In perennial ryegrass (*Lolium perenne* L.) the only PAs that have been identified are the thesinine-rhamnoside group, which displays significant genetic variation. Homospermidine synthase (HSS) has evolved from deoxyhypusine synthase (DHS) and catalyses the first step in the PA pathway, making it a key candidate for the investigation of genes influencing observed PA trait variation.

**Results:**

During PCR amplification and sequence analysis of DHS we identified two putative HSS genes in perennial ryegrass. One of the genes (*LpHSS1*) was absent in some perennial ryegrass plants. Thesinine-rhamnoside levels were measured using liquid chromatography coupled with mass spectrometry in a diverse association mapping population, consisting of 693 plants free of fungal endophytic symbionts. Association tests that accounted for population structure identified a significant association of absence of the *LpHSS1* gene with lower levels of thesinine-rhamnoside PAs. HSS-like gene sequences were identified for other grass species of the Poaceae, including tall fescue, wheat, maize and sorghum.

**Conclusion:**

HSS is situated at the crucial first step in the PA pathway making it an important candidate gene for investigation of involvement in PA phenotypic variation. In this study, PA level in perennial ryegrass was strongly associated with the presence or absence of the *LpHSS1* gene. A genetic marker, developed for the presence/absence of *LpHSS1*, may be used for marker-assisted breeding to either lower or increase PAs in breeding populations of perennial or Italian ryegrass to investigate a potential role in the deterrence of herbivore pests. The presence of HSS-like genes in several other Poaceae species suggests that PA biosynthesis may occur in plant family members beyond perennial ryegrass and tall fescue and identifies a potential route for manipulating PA levels.

**Electronic supplementary material:**

The online version of this article (10.1186/s12870-018-1269-6) contains supplementary material, which is available to authorized users.

## Background

Pyrrolizidine alkaloids (PAs) are a diverse class of secondary metabolites produced by plants to deter herbivores [1, 2]. PAs are produced mainly in angiosperms and are predominantly found in species belonging to the Boraginaceae, Asteraceae, Fabaceae and Orchidaceae families [3, 4]. For several plant families PAs are notorious for their toxicity to livestock [5, 6] and occasionally humans, through use as herbal supplements [7] and via food contamination [8]. The lolines, produced by symbiotic endophytic fungi (*Epichloe* spp.), are one of the most well researched groups of PAs found in forage grasses [9]. Lolines are beneficial to the host plant by deterring a variety of insect pasture pests [10–12]. In contrast, little is known about plant-derived PAs in grasses and the role they could play as defence compounds. During the analysis of perennial ryegrass (*Lolium perenne* L.) metabolites, several abundant novel alkaloids were identified as ions using direct infusion mass spectrometry [13] and later characterised as the PAs E-thesinine-O-4′-α-rhamnoside, Z-thesinine-O-4′-α-rhamnoside and their glycoside derivatives [14]. This was the first time plant-derived PAs were identified in a grass from the Poaceae family. Whether these thesinine-rhamnosides play a role in deterring herbivores or have an alternative function is currently unknown.

Homospermidine synthase (HSS) catalyses the first major step in the PA pathway by transferring an aminobutyl group from spermidine to putrescine to form homospermidine. Homospermidine is then incorporated into the necine base which is the common moiety to all PAs. HSS genes have evolved from a duplication of deoxyhypusine synthase (DHS) [15] which is involved in the post-translational activation of eukaryotic initiation factor 5A (eIF5A) and is essential for cell proliferation [16]. This reaction is highly conserved across eukaroytes, and DHS and HSS genes have been well characterised in orchids [17] and several other angiosperms [18]. HSS differs from DHS in that it has lost its ability to bind and modify the eIF5A precursor protein [19].

Perennial ryegrass is a diploid outcrossing, self-incompatible species that is used extensively as a forage grass in temperate agricultural zones as well as being widely employed as an amenity grass. Breeding populations are highly heterogeneous, providing several challenges to breeders selecting for complex quantitative traits and to researchers applying marker-assisted selection [20]. Several large effect quantitative trait loci (QTL) have been identified in an F_1_ perennial ryegrass mapping population for some of the major alkaloids, including the E/Z-thesinine-rhamnoside PAs [21]. QTL mapping is often imprecise, identifying a large chromosomal region with numerous possible causative genes underlying the trait. Candidate-gene based association mapping [22] is one approach that can be subsequently used to identify the causative genes underlying trait variation, especially where strong evidence exists for the candidate gene from pathway biochemistry or mutational analysis. Rapid linkage disequilibrium (LD) decay is beneficial for high resolution association mapping to identify the gene and underlying functional nucleotide polymorphisms responsible for trait variation [23]. Linkage disequilibrium (LD) in perennial ryegrass rapidly decays for most genes, with r^2^ values of 0.2 within 500 bp [24, 25], where the population represents adequate diversity of the species. Consequently, several candidate gene-based association mapping studies have been performed in perennial ryegrass for a range of traits, including flowering time [26], drought tolerance [25] and fructan content [27].

In this study we analysed a diverse association mapping population and detected significant variation in thesinine-rhamnoside PAs and discovered that a homospermidine synthase gene (Lp*HSS1*) was absent in some of the perennial ryegrass plants. To determine whether this polymorphism was responsible for variation in PA content we performed association tests that accounted for population structure. Significant associations were detected between low levels of the thesinine-rhamnoside group of PAs and the absence of the Lp*HSS1* gene (*HSS1-*). This provides an opportunity to determine the function of these plant PAs in ryegrass. Putative *HSS* genes identified in tall fescue, wheat, sorghum and maize may potentially enable the manipulation of PA levels in these other grass species.

## Methods

### Plant material

The association mapping population included 693 diverse individuals derived from a range of elite breeding populations, cultivars and wild ecotypes of perennial ryegrass (Additional file 1). All population entries were heat and fungicide-treated, prior to the trial, to remove fungal endophytic symbionts (*Epichloë festucae* var. *lolii*). This was necessary to eliminate variation in endophyte strain or endophyte status (infected vs. not infected) as potentially confounding factors in downstream analyses. Successful removal of endophyte was confirmed in each individual using tiller immuno-detection blots [28]. Sixteen plants from various Italian ryegrass cultivars (*Lolium multiflorum* Lam.) (Additional file 2) were also used to extract DNA for marker testing. Plants from the association mapping population were grown outdoors in 1.5 L pots filled with a commercial sand-peat potting mixture, at AgResearch Grasslands, Palmerston North (latitude 40.3523° S, longitude 175.6082° E), New Zealand. Each genotype was clonally replicated five times in a repeated (*n* = 5 replicate blocks) randomised row-column design which was constrained to avoid clonal replicates being present more than once in the same row or column. Plants were cut back every 3 weeks to simulate rotational grazing and plant material (predominantly consisting of the leaf blades) was harvested to a level of 40 mm above ground in May 2012 (Southern hemisphere late autumn).

### LC-MS analysis of thesinine-rhamnoside content

Plant material was immediately placed into liquid nitrogen, freeze dried and ground to a fine powder in a laboratory sample mill. Fifty mg of ground plant material was weighed into a 2.0 mL micro-centrifuge tube along with one 3 mm zirconium bead to enhance extraction. One mL of extraction solvent (50% acetonitrile:50% water, 0.1% formic acid, containing 10 μg/mL dichloroflourescein and d_2_-tyrosine as internal standards) was added and the tube was capped, shaken for 1 min (Qiagen Tissue Lyser II) and then centrifuged in a Minifuge at 14,000 g for 3 min. Approximately 500 μL of the supernatant was placed in a 2 mL glass autosampler vial, capped and stored at − 20 °C until analysis. Samples were analysed by reverse phase (RP) LC-MS using an Agilent RRHD SB-C18 column and Thermo Exactive mass spectrometer with ESI in positive mode. The resulting data were subjected to peak detection process with PhenoAnalyzer (SpectralWorks Ltd., Manchester, UK), using the key data collection and processing parameter settings described previously [29]. The resulting peak data matrices underwent a series of quality control filtering before statistical analysis was conducted to probe into biological effects. These filtering steps, including missing value imputation, peak merge and deisotoping, run-order correction and batch effect corrections were accomplished using in-house scripts (available upon request). The peak intensity of four targeted metabolites were selected for this investigation. These are E-thesinine-rhamnoside (m/z 434.2159, RT 4.51), Z-thesinine-rhamnoside (m/z 434.2159, RT 4.73), E-thesinine-rhamnoside-glycoside (m/z 596.2681, RT 4.33), and Z-thesinine-rhamnoside-glycoside (m/z 596.2681, RT 4.55).

### Statistical analysis of thesinine-rhamnoside traits

Thesinine-rhamnoside signal intensity data were analysed using the variance component analysis procedure, residual maximum likelihood (REML) option, in GenStat [30]. A completely random linear model was used in the analysis using the REML algorithm. The final, adjusted genotypic means were based on Best Linear Unbiased Predictors (BLUP’s). Estimation of the significance of the genotypic variance component for each trait was based on the log-likelihood ratio test. The variance components generated from the REML analysis were used to estimate clonal mean repeatability (R_c_) [31], for the four metabolites, using the model R_c_ = σ^2^_g_/(σ^2^_g_ + σ^2^_ε_/n_r_), where; σ^2^_g_ is the genotypic component of variance, σ^2^_ε_ the residual variance and n_r_ is the number of replications. Pearson correlation coefficients amongst traits were obtained using GenStat [30]. BLUP’s were square root and log-transformed to assess the effect on association tests.

### PCR marker development and linkage mapping

Two perennial ryegrass expressed sequence tags (ESTs) previously submitted to GenBank (GR522183 and GR514484) were identified as *LpDHS* and *LpHSS1* and plasmid clones fully sequenced in the forward and reverse directions using Sanger sequencing. A third gene (*LpHSS2*) was identified during PCR amplification of the initial genes*.* All three perennial ryegrass genes are annotated according to function which has been validated by in vitro protein assays (Dietrich Ober, pers. comm). Genomic sequences were sourced from inhouse gene-enriched Genethresher sequences [32] and several gaps filled by the cloning and sequencing of PCR products (Additional file 3). PCR products were cloned into p-GEM-T (Promega) according to manufacturer’s directions. Colony PCR’s were performed on at least eight clones, purified using ExoSAP-IT (Applied Biosystems) according to manufacturer’s directions and Sanger sequenced. Sequences were assembled and consensus derived using the software Sequencher v4.10.1 (GeneCodes Corporation). Genomic DNA sequence and inferred protein sequences were deposited in GenBank for *LpDHS* (MF375394), *LpHSS1* (MF375393) and *LpHSS2* (MF375392). PCR primers specific to the Lp*HSS1* gene (Additional file 3) were used to test for the presence of the gene. Genomic DNA extractions for the entire association population and 16 Italian ryegrass plants were performed using the DNAeasy plant maxi kit (Qiagen) following the manufacturer’s recommended protocol. PCR amplification was performed in a total volume of 20 μl containing 20 ng of genomic DNA, 1.5 mmol/L MgCl_2_, 0.5 mmol/L dNTPs, 1 U Platinum *Taq* polymerase (Invitrogen), 1× Invitrogen PCR buffer, and 0.5 μmol/L primer. Primers F5, F9 and R8 were selected for evaluating the entire association mapping population for the presence or absence of *LpHSS1* in a multiplex PCR reaction. In the multiplex reaction F9 and R8 primers were at 0.5 μmol/L and F5 at 0.125 μmol/L. To ensure a negative result for *LpHSS1* was not a consequence of a failed PCR, assays were also multiplexed with an internal control based on the *cab* gene [33] using primers (cab-f ^5’^GTCTCGACTACCTCGGCAAC^3’^ cab-r ^5’^ACCGAACATGGAGAACATGG^3’^) at 0.075 μmol/L each. This internal positive control amplifies an expected product of 254 bp. Thermocycling was performed in a 9700 Gene Amp (Perkin Elmer) PCR machine with an initial cycle of 5 min at 94 °C; 35 cycles at 94 °C for 10 s, 57 °C for 10 s and 72 °C for 1 min; final extension at 72 °C for 5 min. In all other PCR reactions thermocycling was as above except annealing temperature varied as indicated in Additional file 3.

Using MAPMAKER/EXP 3.0 [34] the Lp*DHS1, LpHSS1* and Lp*HSS2* genes were mapped by genetic linkage analysis in an F_1_ population comprised of 94 perennial ryegrass individuals, using gene markers that reveal polymorphisms at the three loci (Additional file 4). Framework SSR markers are reported in a previously published linkage map [32].

### Diversity analysis of DHS and HSS gene sequences

*Festuca arundinacea* (tall fescue) EST sequences were retrieved from Genbank by BLAST using *LpDHS*, *LpHSS1* and *LpHSS2*. After assembly of ESTs into nucleotide consensus sequences protein translations for two tall fescue gene paralogs (FaDHS and FaHSS1) (Additional file 5) were obtained and used for sequence homology and phylogenetic analysis.

Sequences covering a range of Poaceae species were chosen from GenBank for sequence alignment and phylogenetic analysis and are as follows: NP_001051218 (*Oryza sativa japonica* DHS), XP_006650561 (*Oryza brachyantha* DHS), XP_003559916 (*Brachypodium distachyon* DHS), EMT19875 (*Aegilops tauschii* DHS), ACP28133 (*Triticum aestivum* DHS), AER42619 (*Hordeum brevisubulatum* DHS), XP_004981833 (*Setaria italica* DHS), XP_002444933 (*Sorghum bicolor* 07 g001640 DHS), XP_002466487 (*Sorghum bicolor* 01 g008630 DHS), XP_0024446268 (*Sorghum bicolor* 06 g011780 HSS), NP_001149084 (*Zea mays* DHS), NP_001130806 (*Zea mays* HSS), XP_020196793 (*Aegilops tauschii HSS)*. The *Triticum aestivum* HSS was retrieved from the UniProt database (accession A0A1DSYB60). The *Hordeum vulgare* DHS amino acid sequence was obtained from a protein translation of the full length EST sequence AK248438. Two functionally characterised protein sequences were used from the monocot orchid *Phalaenopsis amabilis* CAR31338.1 (HSS), CAL69908.1 (DHS).

Protein and DNA alignments were performed using the Geneious software v7.1.5 (http://www.geneious.com [35]) with the ClustalW alignment option with a BLOSUM cost matrix. Gblocks v0.91b (web-based- http://molevol.cmima.csic.es/castresana/Gblocks_server.html) was used to eliminate poorly aligned positions and gaps using default parameters providing 238 amino acids for final analysis. An unrooted phylogenetic tree of DHS and HSS genes from the Poaceae family was constructed in Geneious using the PhyML (v3.2) plug in using the WAG amino acid substitution model with 1000 replicates to obtain bootstrap proportions.

### Population structure and association testing

A core set of 308 SNP markers from 308 genes (Additional file 6), with minor allele frequency > 0.1 and which were dispersed relatively evenly across the genome (based on synteny with rice), were used for evaluating population structure (Q) and kinship (K). Population structure was assessed using the software STRUCTURE v2.3.4 [36]. The number of subpopulations (*k*) was determined by running *k* from 1 to 10 using the admixture model with a burn in time of 50,000 and 100,000 iterations for each run. Each *k* was run ten times and the software STRUCTURE HARVESTER [37] used to analyse results. The method of Evanno et al. [38] was used to identify the best estimate of *k* and was subsequently used to assign each individual to a Q-matrix. Association tests utilising BLUP’s for the PAs were performed in the software TASSEL version 5.2.9 [39] using GLM (Q) and MLM (Q + K) with the kinship matrix (K) calculated using the centred IBS option (Additional file 7) and the population structure matrix Q described above.

## Results

### Statistical analysis of thesinine-rhamnoside traits

The abundance in perennial ryegrass of the four targeted metabolites, E-thesinine-rhamnoside, Z-thesinine-rhamnoside, E-thesinine-rhamnoside-glycoside and Z-thesinine-rhamnoside-glycoside, were estimated based on peak intensities. Frequency distributions for each trait appeared right-skewed or bimodal (Additional file 8A). Both non-transformed BLUP’s and square root and log-transformed BLUP’s were tested in association analyses but all led to similar conclusions. Therefore, only results based on non-transformed BLUP’s are described from here on. All four metabolites demonstrated highly significant genotypic variation in the association population (Table 1). R_c_, the upper limit of the degree of genetic determination [31], was also high for all four metabolites (> 0.948, Table 1), which further confirms a strong genetic basis to the observed phenotypic variation. Thesinine-rhamnoside traits were all significantly correlated to each other (*P* < 0.001) as determined by Pearson’s correlation coefficient analysis (Additional file 8B).

**Table 1:** Median metabolite level prediction (BLUP’s), range, genotypic variance component (σ^2^_g_) with associated standard error (SE), significance and progeny clone repeatability (Rc), for four PA traits measured in an association population of 693 perennial ryegrass plants (*n* = 5 clonal replicates)

Metabolite	Median (× 10^5^)	Range (× 10^5^)	σ^2^_g_ ± SE (× 10^11^)	Significance	R_c_
E-Thesinine-rhamnoside	248.1	23.3–1089.8	4986 ± 274.1	*P* < 0.001	0.979
E-Thesinine-rhamnoside-glycoside	27.8	3.8–279.1	259 ± 14.1	*P* < 0.001	0.984
Z-Thesinine-rhamnoside	92.1	13.5–550.7	1150 ± 65.5	*P* < 0.001	0.948
Z-Thesinine-rhamnoside-glycoside	14.8	3.3–204.2	83 ± 4.6	*P* < 0.001	0.962

### Genotyping and linkage mapping

**Fig. 1:**
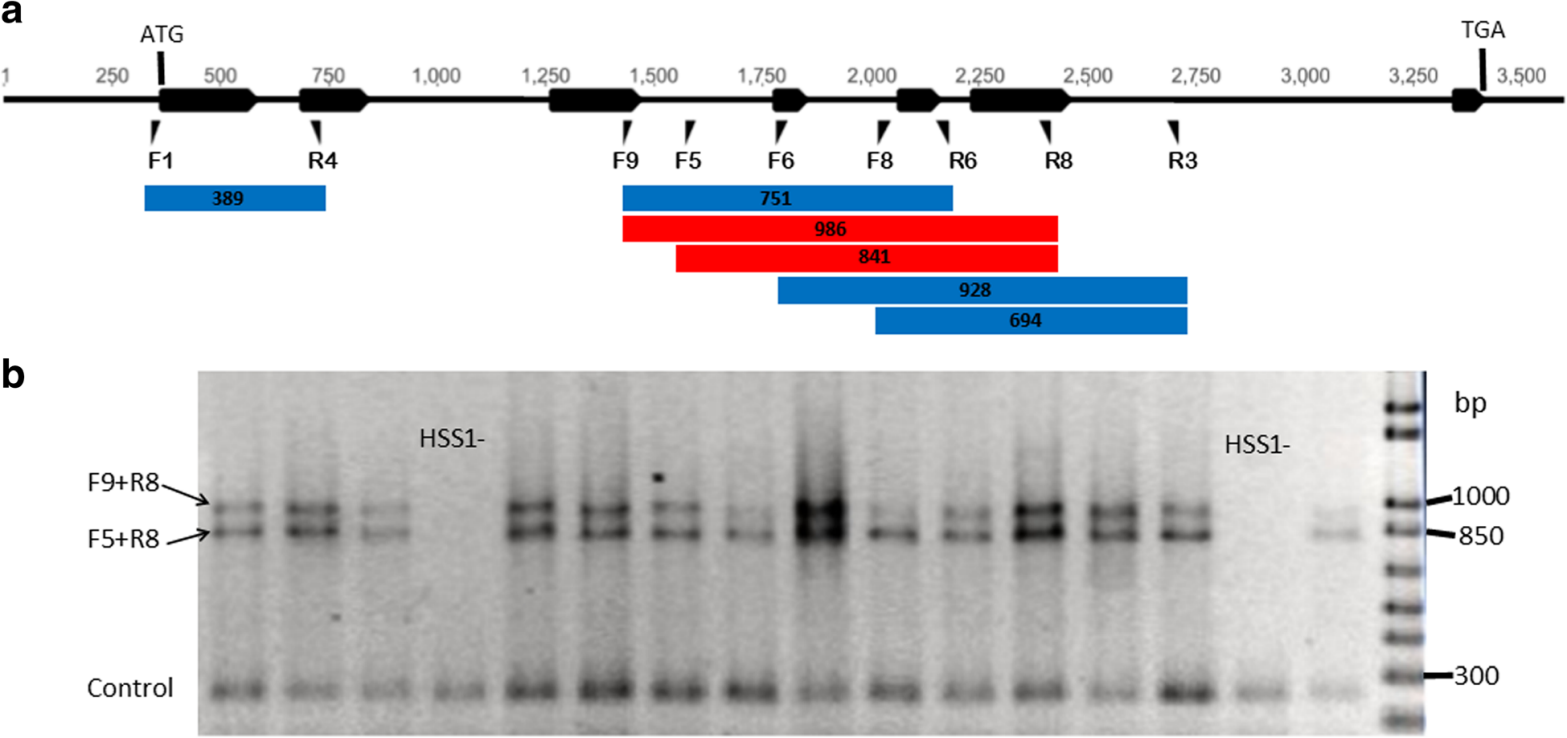
Genotyping assays for the presence (*HSS1+*) or absence (*HSS1-*) of the *LpHSS1* gene*.* **a** Location of primers within the *LpHSS1* gene. Black arrow bars represent exon locations of the *LpHSS1* gene and the position of the start (ATG) and stop (TGA) codons are indicated. Blue bars represent PCR products from various primer combinations used to genotype plants. Red bars represent the F9 + R8 and F5 + R8 PCR products which were developed into a multiplex PCR assay for the genotyping of the entire association mapping population. Numbers given on blue and red bars are PCR product sizes. **b** Screening plants from the association mapping population for *LpHSS1* using the F5 + F9 + R8 multiplex assay. The positive control product from the endogenous *cab* gene confirms a successful PCR when no *LpHSS1* gene product is amplified.

**Fig. 2:**
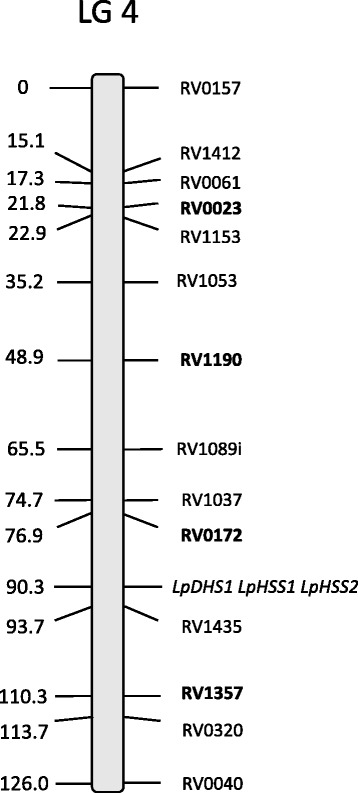
Location of the *DHS* and *HSS* genes on linkage group 4 in perennial ryegrass, determined by genetic linkage analysis. Map distance in centimorgans is shown at the left and marker names to the right. Bold markers represent framework SSR markers [32]

A panel of 16 diverse perennial ryegrass plants from the association mapping population were used to validate the designed primer sets within the *LpHSS1* gene (Fig. 1a). Two plants failed to amplify the Lp*HSS1* gene across all 6 primer sets tested (blue and red bar regions depicted in Fig. 1a). The plants that amplified PCR products gave positive reactions for all 6 primer sets. In a similar survey of 16 plants from nine Italian ryegrass cultivars, plants from six cultivars failed to amplify the *LpHSS1* gene with all six primer sets (Additional file 2). Two primers (F5 + F9) were multiplexed with primer R8 (Fig. 1a and b) and a positive control primer set (to detect failed reactions) and used to genotype the full association mapping population for the presence (*HSS1+*) or absence (*HSS1-*) of the *LpHSS1* gene. *HSS1+* plants can either be homozygous or hemizygous for the *LpHSS1* gene. The incidence of *HSS1-* across the entire association mapping population was 0.18. *HSS1-* was at a much higher incidence in some populations than others (Additional file 1). In a relatively restricted sample of wild ecotypes, *HSS1-* was observed in material sourced from regions as diverse as Greece, Italy, Tunisia and Uzbekistan. *HSS1-* was observed in many European cultivars (19/39) but not identified in any of the Australian cultivars and only 3 out of 18 New Zealand cultivars. Two of the New Zealand cultivars have a high incidence of *HSS1-* (Tolosa 8/13 plants; Grasslands Impact 2/4 plants). Genetic linkage analysis of the *LpDHS*, *LpHSS1*, and *LpHSS2* mapped all genes from perennial ryegrass to the same location on the distal half of linkage group (LG) 4 (Fig. 2).

### Diversity Analysis of *DHS* and *HSS* gene sequences in the Poaceae

**Fig. 3:**
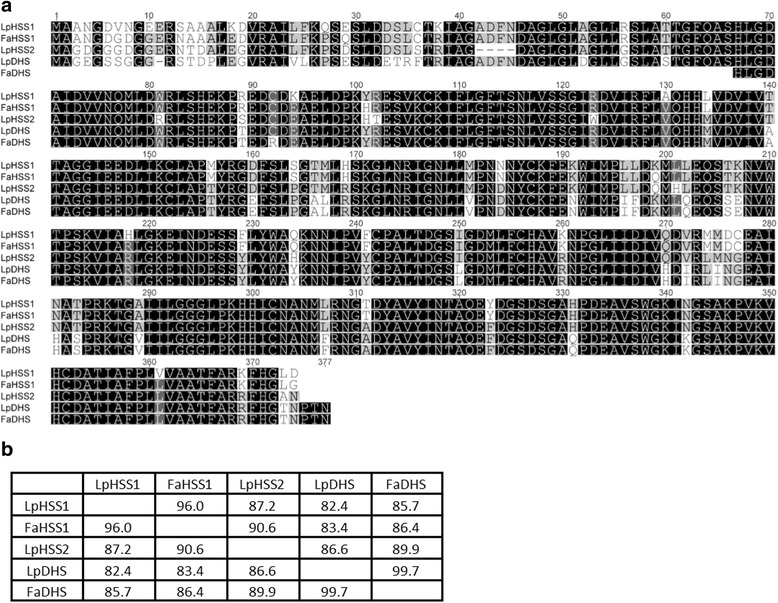
Amino acid sequence similarity of DHS and HSS proteins from perennial ryegrass and tall fescue. **a** ClustalW alignment of amino acid sequences. **b** Amino acid percentage similarity matrix

**Fig. 4:**
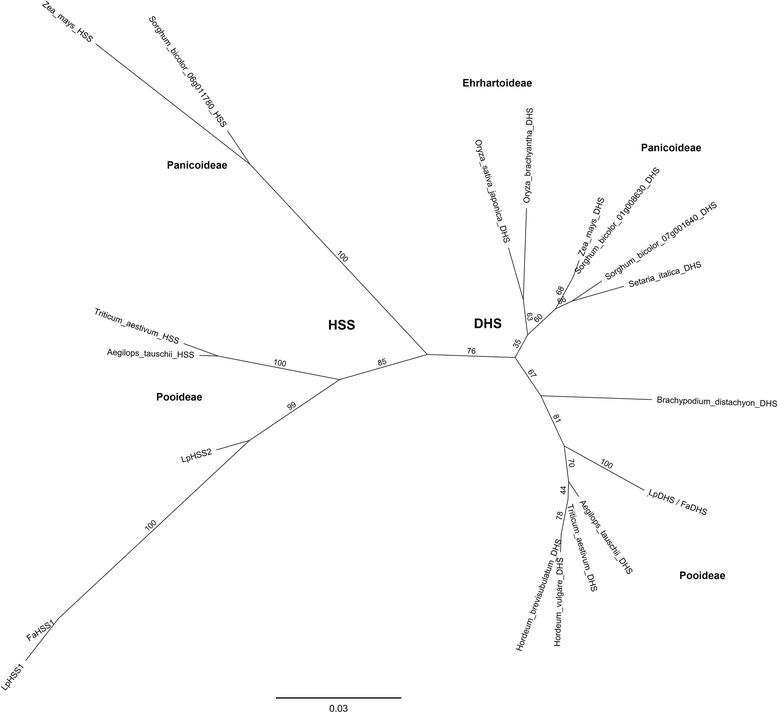
Unrooted maximum likelihood tree of DHS and HSS amino acid sequences of various Poacae species, including *Lolium perenne* (Lp) and *Festuca arundinacea* (Fa). Bootstrap proportions resulted from 1000 replicates. Bar represents 0.02 amino acid substitutions per site

**Fig. 5:**
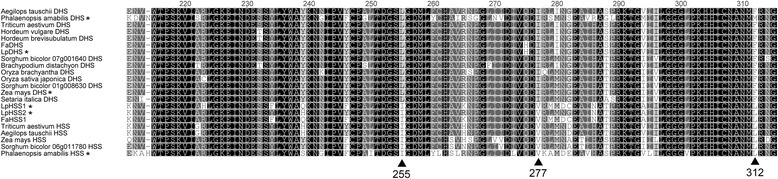
Amino acid alignment in a conserved region of the DHS and HSS proteins of Poaceae and Phalaenopsis species. Amino acids indicated with triangles are conserved across the HSS-like proteins. Asterisk indicates functionally validated proteins

Orthologs of the *LpDHS* and *LpHSS1* genes in tall fescue shared an amino acid identity of 97.7 (*FaDHS*) and 96.3% (*FaHSS1*) respectively (Fig. 3a and b). No tall fescue sequences for *LpHSS2* were identified in GenBank. Sequences from Poaceae species were putatively assigned function as DHS or HSS based on combined lines of evidence. Genes were assigned as HSS’s if the sequence was divergent from the main DHS phylogenetic clade, they contained conserved amino acid changes known to favour HSS activity [40], and if a second gene that resided in the DHS phylogenetic clade was present. Species with a single gene present from a sequenced genome also supported assignment as DHS. From the phylogenetic tree (Fig. 4) most Poaceae amino acid sequences cluster with DHS in respective subfamilies. However, both wheat, *Aegilops tauschii (ancestor of wheat)*, maize and sorghum possessed sequences divergent from the main DHS clade, as observed for Lolium/Festuca HSS’s, suggesting they may function as HSS’s. In one particular key region of these two proteins, several amino acid differences (255, 277 and 312) are conserved with HSS-like sequences compared to DHS sequences in Poaceae (Fig. 5). The valine 277 is also conserved in a homospermidine synthase functionally characterised in orchids (Fig. 5) [17].

### Population structure

The software STRUCTURE HARVESTER was used to analyse output from STRUCTURE (testing *k* = 1–10) to determine that the optimal number of population subgroups within the association mapping population was three (Additional file 9). The three subpopulations reflected geographical origins to some extent. Group 1 was comprised of cultivars developed in Europe, natural populations sourced from Europe and the majority of cultivars from New Zealand and Australia. Group 2 mainly identified North African–sourced populations and New Zealand breeding populations with a known North African contribution (Dr Alan Stewart, pers. comm). The third group principally included New Zealand cultivars and breeding populations with a recognised basis in germplasm sourced from Northwest Spain (e.g. Tolosa and Impact cultivars) [41] and derived breeding populations (Dr Alan Stewart pers. comm).

### Association of *HSS1-* with thesinine-rhamnoside levels

**Fig. 6:**
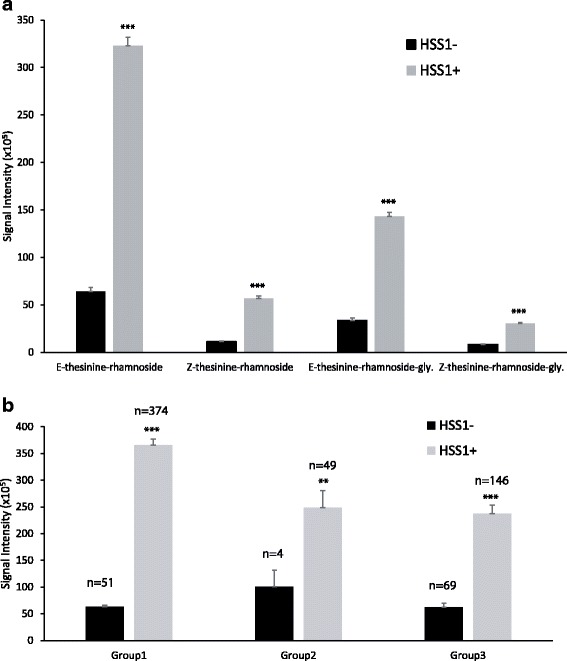
**a** Effect of *HSS1-* on the content of different thesinine-rhamnosides in the association mapping population of 693 plants. **b** Effect of *HSS1-* on E-thesinine-rhamnoside content in different population subgroups as identified by the programme STRUCTURE. ** and *** indicate significance at *P*<0.01 and *P*<0.001 respectively. Error bars represent standard error of the mean

Statistical tests were performed for the association of thesinine-rhamnoside PA levels with the presence or absence of the *LpHSS1* gene. Significant associations were detected for all four thesinine-rhamnoside PAs using either the MLM (Q + K) or GLM method (Q) implemented in the software TASSEL (Table 2). The strongest association using MLM analysis, which corrects for population structure and kinship, was for E-Thesinine-rhamnoside (*p* = 1 × 10^− 17^) with a marker R^2^ value of 0.110. The E/Z-thesinine glycosides had higher *p* values (8.5 × 10^− 13^ and 2.9 × 10^− 10^) and lower R^2^ values (0.076 and 0.059) but were still highly significant associations. The *HSS1-* genotype has a large effect on reducing thesinine-rhamnoside content when compared to plants that possess the *HSS1+* allele in the association population (Fig. 6a). For example, a 5-fold difference in E-thesinine rhamnoside levels occurred between *HSS1-* and *HSS1+* plants (Fig. 6a). Decreased thesinine-rhamnoside levels in *HSS1-* plants is also evident when analysed by subpopulation (Fig. 6b).

**Table 2:** Association of *HSS1-* with level of thesinine-rhamnosides using GLM (Q) and MLM (Q + K) analysis

Pyrrolizidine alkaloid	GLM		MLM	
	*p*-value	R^2^ marker	p-value	R^2^ marker
E-thesinine-rhamnoside	7.7 × 10^− 33^	0.174	1.0 × 10^−17^	0.110
Z-thesinine-rhamnoside	3.9 × 10^−25^	0.134	7.5 × 10^−14^	0.083
E-thesinine-rhamnoside-glycoside	1.1 × 10^−21^	0.122	8.5 × 10^−13^	0.076
Z-thesinine-rhamnoside-glycoside	2.9 × 10^−16^	0.090	2.9 × 10^−10^	0.059

## Discussion

Plant-derived PAs have been identified and studied mainly in angiosperm species [3, 4] and less so in the commercially important grasses of the Poaceae family where they have only been identified in a few *Lolium* and *Festuca* species [14]. In this study, the identification of a homospermidine synthase gene (*LpHSS1*) that is present/absent in a perennial ryegrass association mapping population has allowed us to determine that the *LpHSS1* gene is actively involved in the biosynthesis of PAs and is partially responsible for phenotypic variation of PA levels.

The *DHS* and *HSS* genes of perennial ryegrass were mapped using genetic linkage analysis to the distal half of linkage group LG4. All 3 genes are co-located (Fig. 2) indicating that they most likely evolved through tandem duplication. Their map location is not in the same region as large effect QTLs identified previously for thesinine-rhamnoside PAs [21], which were proximally located in LG4. Other genes residing further down the PA pathway or transcriptional regulator genes not yet identified may explain the earlier identified QTLs.

The majority of New Zealand perennial ryegrass cultivars originate from a narrow base of two ecotypes (Hawkes Bay and Mangere) [41] which is the likely reason for the low incidence of *HSS1-* in this germplasm source. The New Zealand cultivars Tolosa and Grasslands Impact, have a notably high incidence of *HSS1-* attributed to introgression of Northwest Spain germplasm [41]. The further introduction of NW Spain and other European germplasm into New Zealand breeding populations [41] may disperse *HSS1-* more widely. The relatively wide-spread dispersal of *HSS1-* within perennial ryegrass and Italian ryegrass indicates that this locus is not essential and probably incurs no major penalty to fitness.

The three subpopulations (k) identified in this study were similar to the subpopulations determined in an earlier perennial ryegrass association mapping study [42]. In both these and other studies [25, 43] the main subpopulation group identified is from Europe, New Zealand and Australia and represents the majority of commercial cultivars and elite breeding populations. The association mapping population in our study was biased towards this major subpopulation group so as to identify markers in commercially relevant breeding populations. Wild ecotypes from Africa and Europe are less adapted to the New Zealand environment, with a high mortality rate. As a consequence our association population did not cover the full diversity of the species and could explain why several minor subpopulation groups reported in another study [25] were not detected here. Typically candidate gene-based association mapping approaches rely on testing multiple SNPs that tag gene haplotypes in the hope of identifying an underlying functional nucleotide polymorphism (FNP) or one in LD to the FNP responsible for trait variation [22]. In this study the presence/absence of a gene among individuals from a population at a relatively high incidence allowed us to quickly evaluate whether the gene was responsible for trait variation avoiding an initial laborious SNP discovery and haplotype determination phase. For the presence/absence of the *LpHSS1* gene the effect on level of thesinine-rhamnosides is relatively large (Fig. 6), as would be expected for a candidate gene at the crucial first step of the pathway. This strong functional association is substantiated by the relatively large-sized population (693 plants) coupled with the very small *p*-values obtained (< 6 × 10^− 10^) for marker-PA associations. Marker p-values using GLM (Q) analysis were probably inflated compared to MLM (Q + K) which additionally accounts for kinship within the population and results in improved control of type I and type II error rates [44, 45]. Reduced levels of PAs in *HSS1*- plants were found for all 3 sub-population groups identified (Fig. 6b) providing additional assurance that associations are not falsely attributed to population structure effects.

In most species a single functional DHS gene exists [15]. Of interest is the identification of two sequences from sorghum that are relatively similar to each other and grouped with other DHS genes. The sorghum protein from locus 07 g001640 appears truncated and a result of an incorrect protein prediction as exon1 and exon2 are present in the genome sequence. If this locus encodes a functional gene it would be of interest to determine whether both sorghum genes can function as DHS as has been found in the species *Crotalaria juncea* [46]. In species that produce PAs, the DHS gene has been duplicated and recruited as a HSS [17, 46]. When analysing sequences of the Poaceae family wheat, maize and sorghum were found to possess sequences that had diverged significantly from DHS. These divergent sequences, as identified in perennial ryegrass and tall fescue, are more likely to function as HSS but require functional validation to be certain. Further evidence for their role as HSSs includes several amino acid changes that are different when compared to DHS gene sequences in Poaceae and extend beyond this family to a number of angiosperm plant families [40]. One of the key amino acid differences when comparing HSS sequences across species is amino acid number 277 (valine) (Fig. 5), which has been shown to favour the HSS reaction substrate putrescine rather than the DHS substrate eIF5A [40]. No PAs have been identified or reported from maize or sorghum but now the identification of putative HSS genes from these two species suggests that a range of undiscovered PAs could be present. Given the absence of the *LpHSS1* gene regularly observed in perennial ryegrass and pseudogenes detected in other plant species [17, 40, 46] naturally occurring mutations in HSS genes could explain PA variation in plants more broadly, given their key location in the PA pathway.

After a gene duplication, as in the case of HSS from DHS, pseudogenization can occur as two genes of identical function will not be stabilised in the genome [47] unless an additional product of the gene is advantageous. In the case of HSS in many plant species, the gene has undergone adaptive evolution to be recruited into a new role of herbivore defence through PA production [18, 19]. Symbiotic *Epichloё* fungal endophytes of perennial ryegrass [11] produce PAs and a range of other alkaloids, which can be strain specific [48], and which deter various insect pests. More effective host-endophyte relationships might contribute to functional redundancy of HSS genes and thereby hasten the pseudogenization process, resulting in eventual elimination from the perennial ryegrass genome.

Specialist plant insect herbivores have adapted to PAs through various methods of detoxification [2]. PAs introduced into grass plants from symbiotic endophytic fungi on the other hand have proven to be successful as feeding deterrents to invertebrates in several pasture grasses [10, 12]. It is currently unknown whether the thesinine-rhamnoside PAs and others as yet unidentified in perennial ryegrass play a role in insect deterrence. It would be of interest to select families of plants that possess the full range of concentrations of these particular metabolites to investigate feeding response by a range of insects. PAs from some plant species are also renowned for their toxicity to mammalian livestock, where livers can be damaged by pyrrols that are released as breakdown products [6, 49]. Livestock that forage on widely-used pasture grasses, such as perennial ryegrass, are likely to have adapted to the specific assortment of plant defensive PAs ingested, which are presumably negligible in toxicity, with detoxification by rumen bacteria as one potential mechanism of adaption [50]. If plants are bred for extremely high levels of thesinine-PAs, livestock feeding trials to evaluate the potential for any adverse effect should be conducted. The marker developed for the *LpHSS1* gene will facilitate the selection of high and low PA plants to investigate whether it is beneficial to increase levels to deter insect pests or reduce levels if any adverse effect occurs in livestock.

In this study we investigated the thesinine-rhamnoside PAs as these are the only perennial ryegrass PAs identified to date. As the metabolome of perennial ryegrass is further characterized, other PAs of interest will inevitably be identified. Given that the *LpHSS1* gene is at a crucial step in the beginning of the PA pathway it is likely that *HSS1-* genotypes will similarly reduce the levels of any other PAs. The conclusive results of the *LpHSS1* gene being implicated in PA variation suggests future investigation into the *LpHSS1*, *LpHSS2* and *LpDHS* genes for FNPs associated with PA levels is a worthwhile pursuit.

## Conclusions

In this study we have identified a *HSS* gene that is absent in a diverse range of perennial and Italian ryegrass populations. The absence of the gene is strongly associated with reduced levels of the thesinine-rhamnoside group of PAs in perennial ryegrass. The presence of *HSS*-like genes in several other Poaceae species provides evidence that PA biosynthesis may not be limited to perennial ryegrass and tall fescue within this plant family and identifies a potential route for manipulating PA levels.

## Additional files

Additional file 1: Perennial ryegrass germplasm identity and association mapping data. Includes plant number, cultivar/breeding population name, country origin, population type, Margot Forde germplasm accession number, thesinine-rhamnoside BLUPs, presence/absence of HSS1 polymorphism and population structure Q values. (XLSX 79 kb)

Additional file 2: Italian ryegrass cultivars tested for the presence/absence of the Lp*HSS1* gene using six PCR primer sets (See Fig. 1a and Additional file 3 for primer location and sequence). (PDF 437 kb)

Additional file 3: Primer sequences used for PCR and sequencing of the DHS and HSS genes. (XLSX 13 kb)

Additional file 4: Genetic markers used for linkage mapping of the *LpDHS*, *LpHSS1* and *LpHSS2* genes in a F_1_ population of perennial ryegrass. (PDF 428 kb)

Additional file 5: Tall Fescue FaDHS and FaHSS1 translated protein sequences derived from ESTs retrieved from GenBank. (FASTA 1 kb)

Additional file 6: Information for 308 SNPs used for population structure analysis of the perennial ryegrass association mapping population. (XLSX 28 kb)

Additional file 7: Kinship matrix of the association population calculated using 308 SNP markers and the TASSEL kinship centred IBS option. (XLSX 5357 kb)

Additional file 8: Statistical analysis of thesinine-rhamnoside traits in perennial ryegrass. A) Frequency distribution of untransformed thesinine-rhamnoside BLUP’s. B) Pearson correlation coefficients among levels of thesinine-rhamnoside PAs. (PDF 537 kb)

Additional file 9: STRUCTURE HARVESTER results used to determine number of subpopulations (*k*) in the perennial ryegrass association mapping population. (PDF 494 kb)

## Abbreviations

DHS: Deoxyhypusine synthase
ESTs: Expressed sequence tags
FNP: Functional nucleotide polymorphism
GLM: General linear model
HSS: Homospermidine synthase
LC-MS: Liquid chromatography-mass spectrometry
LD: Linkage disequilibrium
LG: Linkage group
MLM: Mixed linear model
PA: Pyrrolizidine alkaloid
QTL: Quantitative trait loci
SSR: Simple sequence repeat
