# Supplementary material for: Low pyrrolizidine alkaloid levels in perennial ryegrass is associated with the absence of a homospermidine synthase gene
Source: BMC Plant Biol. 2018 Apr 6;18:56. doi: 10.1186/s12870-018-1269-6 (PMC5889531; doi:10.1186/s12870-018-1269-6)
Supplement: Supplementary file 2 — Italian ryegrass cultivars tested for the presence/absence of the LpHSS1 gene using six PCR primer sets (See Fig. 1a and Additional file 3 for primer location and sequence). (PDF 437 kb) [file 12870_2018_1269_MOESM2_ESM.pdf]

Additional file 2

Italian ryegrass cultivars tested for the presence/absence of the *LpHSS1* gene using six PCR primer sets (see Figure 1a and additional file 3 for primer location and sequence).

Plant #	Cultivar	Accession (Margot Forde)	*HSS1 presence/absence*[1]
1	Sikem	B 3791	+
2	Sikem	B 3791	-
3	Paloma	B 3812	-
4	Metro	B 3820	-
5	Metro	B 3820	-
6	Matador	B 3817	-
7	Matador	B 3817	-
8	Kitti	B 3792	+
9	Fastyl	B 3786	+
10	Fastyl	B 3786	-
11	Ruten	B 3787	-
12	Ruten	B 3787	+
13	Lemtel	B 3798	+
14	Lemtel	B 3798	+
15	L17 Asso diploid	B 3799	+
16	L17 Asso diploid	B 3799	+

[1] All six primer sets tested for each plant gave the same result.
