# Supplementary material for: Low pyrrolizidine alkaloid levels in perennial ryegrass is associated with the absence of a homospermidine synthase gene
Source: BMC Plant Biol. 2018 Apr 6;18:56. doi: 10.1186/s12870-018-1269-6 (PMC5889531; doi:10.1186/s12870-018-1269-6)
Supplement: Supplementary file 4 — Genetic markers used for linkage mapping of the LpDHS, LpHSS1 and LpHSS2 genes in a F1 population of perennial ryegrass. (PDF 428 kb) [file 12870_2018_1269_MOESM4_ESM.pdf]

Additional File 4

Genetic markers used for linkage mapping of the *LpDHS*, *LpHSS1*, and *LpHSS2* genes in a F1 population of perennial ryegrass

Gene	Forward primer	Forward primer sequence	Reverse Primer	Reverse primer sequence	Polymorphism type mapped
DHS	DHS-f4	gactcgggagcacagccag	DHS-r4	ggtcggatttgtgccatgaaaccta	INDEL
HSS1	HSS1-f8	gcaagactgaagttacactgtgctgactc	HSS1-r8	cggatgtgctcctgaatcactacca	CAPS marker (cut with Pst I)
HSS2	HSSx-f3	ctcgccwccackggrttccagg	HSS2-ASr1	caacttcaatgcacgttgcac	Allele specific dominant marker
