# Supplementary material for: Low pyrrolizidine alkaloid levels in perennial ryegrass is associated with the absence of a homospermidine synthase gene
Source: BMC Plant Biol. 2018 Apr 6;18:56. doi: 10.1186/s12870-018-1269-6 (PMC5889531; doi:10.1186/s12870-018-1269-6)
Supplement: Supplementary file 8 — Statistical analysis of thesinine-rhamnoside traits in perennial ryegrass. A) Frequency distribution of untransformed thesinine-rhamnoside BLUP’s. B) Pearson correlation coefficients among levels of thesinine-rhamnoside PAs. (PDF 537 kb) [file 12870_2018_1269_MOESM8_ESM.pdf]

ADDITIONAL FILE 8

A Frequency distribution of untransformed thesinine-rhamnoside BLUP's

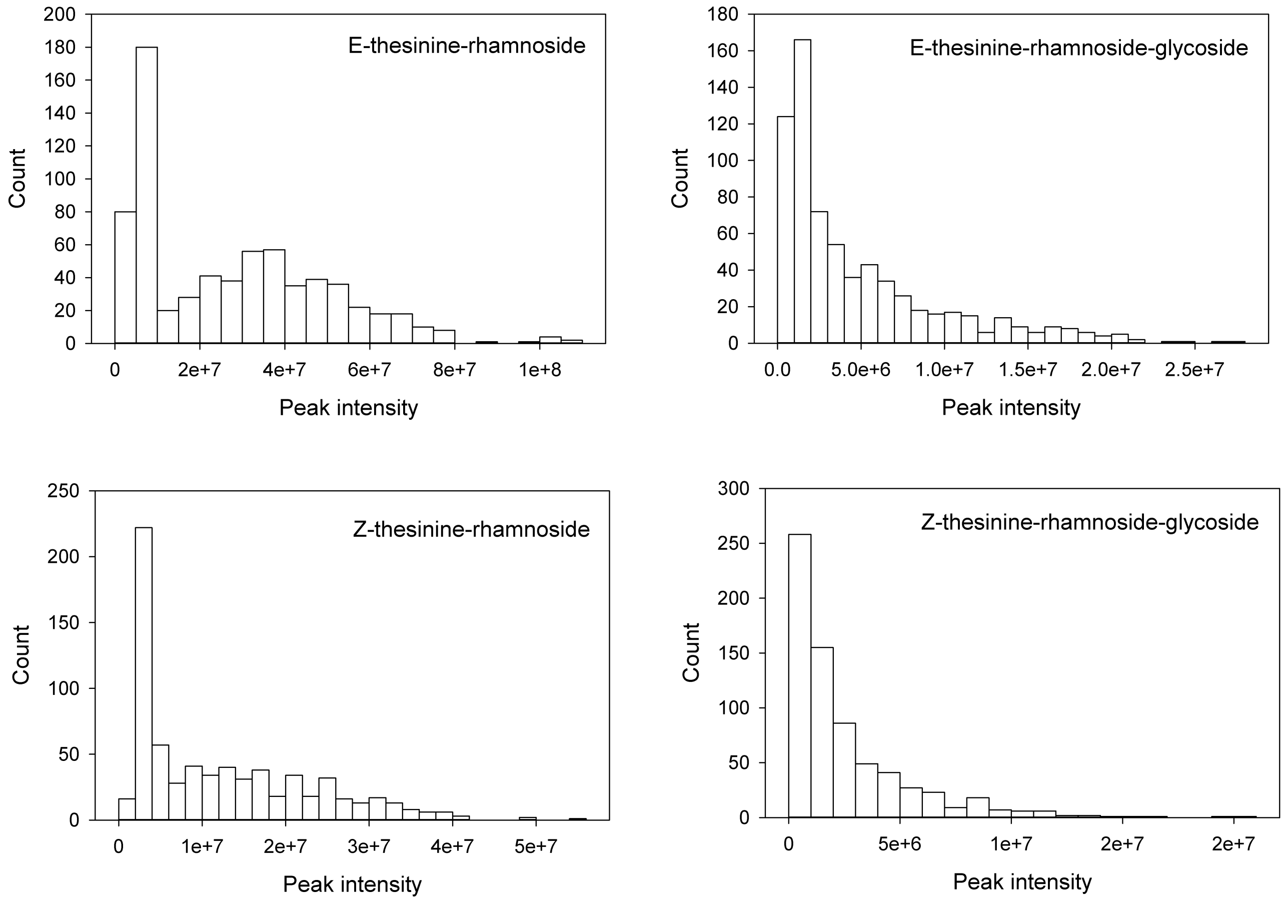

B Pearson correlation coefficients among levels of thesinine-rhamnoside PAs in perennial ryegrass.

	E-thesinine-rhamnoside	E-thesinine-rhamnoside-gly.	Z-thesinine-rhamnoside
E-thesinine-rhamnoside-gly.	0.669***		
Z-thesinine-rhamnoside	0.856***	0.556***	
Z-thesinine-rhamnoside-gly.	0.588***	0.946***	0.561***

*** indicate significance at $P<0.001$
