# Supplementary material for: Low pyrrolizidine alkaloid levels in perennial ryegrass is associated with the absence of a homospermidine synthase gene
Source: BMC Plant Biol. 2018 Apr 6;18:56. doi: 10.1186/s12870-018-1269-6 (PMC5889531; doi:10.1186/s12870-018-1269-6)
Supplement: Supplementary file 9 — STRUCTURE HARVESTER results used to determine number of subpopulations (k) in the perennial ryegrass association mapping population. (PDF 494 kb) [file 12870_2018_1269_MOESM9_ESM.pdf]

ADDITIONAL FILE 9

STRUCTURE HARVESTER output to determine optimal number of subpopulations.

A

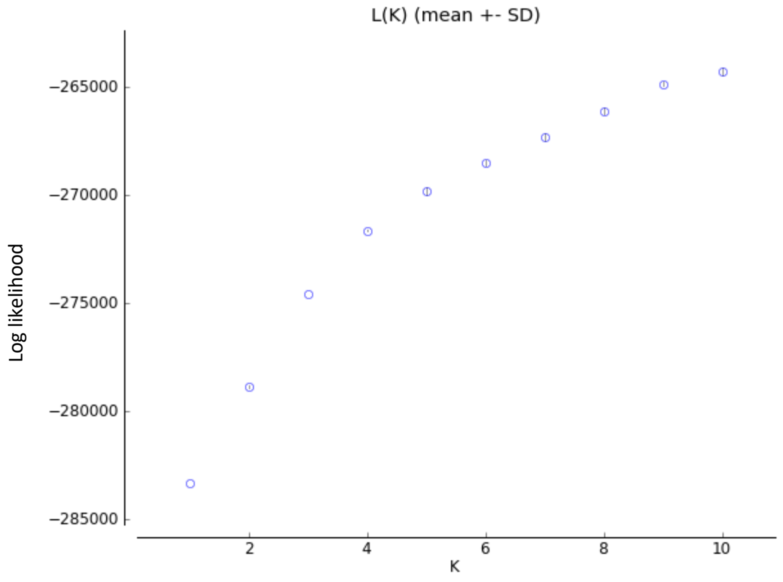

B

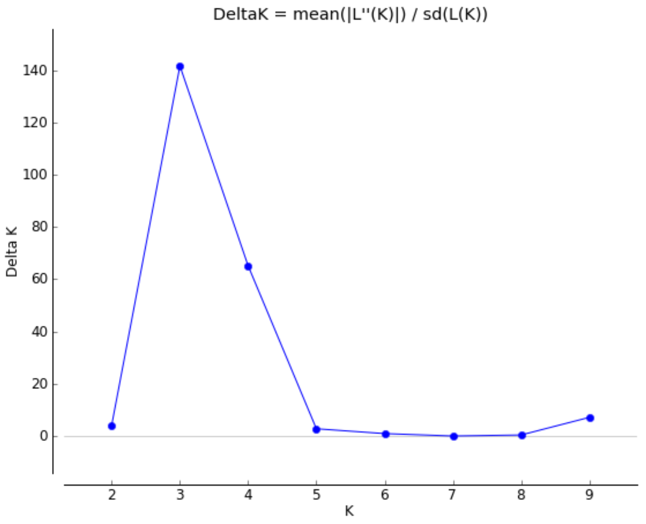

C

K	Reps	Mean LnP(K)	Stdev LnP(K)	Ln'(K)	|Ln''(K)|	Delta K
1	10	-283330.870000	0.437290	—	—	—
2	10	-278873.990000	39.308197	4456.880000	164.260000	4.178772
3	10	-274581.370000	9.621740	4292.620000	1362.580000	141.614712
4	10	-271651.330000	16.868712	2930.040000	1098.790000	65.137753
5	10	-269820.080000	187.508067	1831.250000	527.840000	2.815026
6	10	-268516.670000	109.403017	1303.410000	106.440000	0.972916
7	10	-267319.700000	184.275832	1196.970000	9.780000	0.053073
8	10	-266132.510000	153.725429	1187.190000	66.960000	0.435582
9	10	-264878.360000	95.357062	1254.150000	684.750000	7.180905
10	10	-264308.960000	149.344331	569.400000	—	—

A) Mean log likelihood of K postulated number of subpopulations for K=1 to 10 with bars representing ± standard deviation.

B) Delta K for 1 to 10 subpopulations as calculated by Evanno et al (2005)

C) Table summary of values and highlighted optimal value for number of subpopulations (K)
